# Synchrotron X-ray Absorption Spectroscopy Study of Local Structure in Al-Doped BiFeO_3_ Powders

**DOI:** 10.1186/s11671-019-2965-3

**Published:** 2019-04-18

**Authors:** Turghunjan Gholam, Li Rong Zheng, Jia Ou Wang, Hai Jie Qian, Rui Wu, Hui-Qiong Wang

**Affiliations:** 1 Key Laboratory of Semiconductors and Applications of Fujian Province, Collaborative Innovation Center for Optoelectronic Semiconductors and Efficient Devices, Department of Physics, Xiamen University, Xiamen, 361005 People’s Republic of China; 2 Beijing Synchrotron Radiation Facility, Institute of High Energy Physics, Chinese Academy of Sciences, Beijing, 100049 People’s Republic of China

**Keywords:** Hydrothermal route, X-ray absorption fine structure, BiFeO_3_, Band gap

## Abstract

The Al-doped BiFeO_3_, i.e., BFA_*x*_O powder samples with *x* = 0, 0.025, 0.05, and 0.1, were prepared via the hydrothermal route. The effects of Al substitution on the structural, electrical, and optical properties of BFA_*x*_O samples were investigated. It is found that the substitution of Al ions at B-site of BiFeO_3_ did not cause structural change and it still retains the rhombohedral perovskite structure with *R3c* symmetry, which was confirmed by the X-ray diffraction (XRD) and Raman measurements. X-ray absorption fine structure (XAFS) above the Fe *K*-edge and Bi *L*_3_-edge in BFA_*x*_O powders was also measured and analyzed. Fe ions exhibit mixed valence states (Fe^2+^/Fe^3+^) while Bi ions keep the + 3 valence state in all the samples. Fe *K*-edge XAFS also indicated that there was a competition between hybridization of Fe 3*d* and Al 3*d* with O 2*p* orbitals and occurrence of the more 4*p* orbitals with Al doping. The Bi *L*_3_-edge XAFS revealed that transition from 2*p*_3/2_ to 6*d* state increased, so did the energy of 6*d* state. Besides, Al ion doping affected both the nearest-neighbor and next-nearest coordination shells of Fe atom and nearest-neighbor shells of Bi atom. Ultraviolet-visible (UV-Vis) spectroscopy results show the BFA_*x*_O prepared by hydrothermal method could be an appropriate visible-light photocatalytic material.

## Background

Multiferroics are materials that simultaneously display ferroic properties, such as ferroelectricity, ferromagnetism, and ferroelasticity [1]. Such materials show interesting behaviors such as electrical polarization, which can be controlled with the application of an external electric field or vice-versa. Interest in these materials is owing to their wide applications in chemical biosensors, nanoelectronic and high-density data storage devices, etc [2, 3]. Perovskite structures have the general formula ABO_3_, where O is an ion (refers to oxygen usually) and A and B are cations, respectively. Generally, A-site cation is larger with a lower valence state, which combines with O^2-^ to form a close-packed layer (i.e., at the corner of the unit cell). B-site cation is smaller with higher valence state, which can be adopted into an oxygen octahedral coordination environment (i.e., at the center of an octahedron of oxygen anions) [4]. By definition, almost all the multiferroics are antiferromagnetic (AFM) or weak ferromagnetic (FM) with low transition temperatures. They are divided into two classes: single phase and composite. However, the single-phase multiferroic materials are rarely found in nature which shows the ferroelectric (FE) and FM properties concurrently [5]. Among all the multiferroic materials known today, bismuth ferrite (BiFeO_3_; BFO) is one of the single-phase materials that have a rhombohedral distorted perovskite lattice type with polar space group *R3c*. BFO shows both FE with Curie temperature of *T*_C_ ≈ 1103 k and G-type AFM ordering with Neel temperature of *T*_N_ ≈ 643 k above room temperature (RT) [6]. This material exhibits the AFM G-type spin configuration along the [111]_*c*_ or [001]_*h*_ directions in its pseudo-cubic or rhombohedral structure and has a superimposed spiral spin structure with a periodicity of about 62 Å along the [110]_*h*_ axis at RT [7]. It has a large intrinsic spontaneous polarization of about 90 μC/cm^2^ ascribed to the distortion of octahedral FeO_6_, owing to the presence of 6 *s*^2^ lone pair of electrons [8]. Furthermore, there is a magnetoelectric coupling between FE and magnetic order parameters. FE properties depend on the lone pair electrons, and FM properties rely on the partially filled inner shells, i.e., polarization comes from the Bi-site (A-site), while magnetization comes from the Fe-site (B-site).

Apart from above, unfortunately, the main disadvantages of BFO are its low resistivity or large leakage current, due to the charge defects such as bismuth and oxygen vacancies, impurity phases, valence fluctuations of iron, and poor interfacial quality [9]. In addition, it is difficult to obtain the high-quality BFO because of some impurity phases like Bi_2_Fe_4_O_9_ (space group *Pbam*) and Bi_25_FeO_39_ (space group *I23*). It is inevitable that the impurities are generated in the process of preparation. To address these issues and limitations, several research groups have been using various methods to overcome the defects of BFO, e.g., strain modification, substitution of divalent and rare earth ion doping. Now, within this field of study, with rare earth element or transition metal ion doping at A-site or B-site, or co-doping at A and B-site, the multiferroic properties of BFO can be enhanced. For instance, doping with rare earth element can stabilize the perovskite structure, retain the non-centrosymmetry, and control the vaporization of Bi^3+^ ions [10]. Doping with transition metal ion can reduce the valence fluctuation of Fe^3+^ ions. Elements like Pr, Sm, Eu, Gd, and La [11, 12] for A-site substitution and Mn, Cr, and Ti [13–15] for B-site substitution have already been reported. Moreover, the magnetic, dielectric, and ferroelectric properties can be enhanced with co-doping. For the co-doping of A- and B-sites of BFO, La-Gd, Ba-Ni, Dy-Cr, Y-Mn, and Tb-Ti have been reported [16–20]. Until now, various routes including sol-gel [21], mechanochemical [22], auto-combustion [23], pulsed laser deposition [24]**,** and hydrothermal [25, 26] have been reported to prepare BFO. The hydrothermal method has been widely applied owing to its energy saving, fine dispersion, low cost, and small particle size properties [27]. The reaction temperature should be high enough to form BFO and also used to remove the secondary phases during sample preparation. Most previous studies of Al doped at both A- and B-site of BFO have been investigated for structural, optical, and transport properties by Azam et al [28]. Madhu et al. [29] have reported the photocatalytic applications of B-site Al-doped BFO. Another report from Jawad et al. [30] explored the dielectric behavior of nanostructured BFO ceramics. The work of Wang et al. [31] studied the hollow crystals of Al-doped BFO in detail. However, some important physical properties are still lack of understanding, such as B-site doping effects on the local electronic structure of the materials. At the present stage, X-ray absorption fine structure spectroscopy (XAFS) is one of the powerful ways to study the local environment of an atom and provides structural information of the materials, as well as absorption energy, element valence state, charge transfer, and type of bonding [32]. To the best of our knowledge, there are no found reports on the effect of Al doping at B-site on the local electronic structure of BFO investigation by XAFS.

In this work, the undoped BFO and target compositions BiFe_1-*x*_Al_*x*_O_3_ (BFA_*x*_O) with *x* = 0, 0.025, 0.05, and 0.1 were synthesized via hydrothermal route. The main focus is on the investigation of the effect of Al doping at B-site on the properties of BFO which are compared with undoped BFO. The structural properties were investigated in detail.

## Methods

The hydrothermal method was used to obtain undoped BFO and BFA_*x*_O samples. Chemical reagents used in this work were bismuth nitrate (Bi(NO_3_)_3_·5H_2_O), iron nitrate (Fe(NO_3_)_3_·9H_2_O), aluminum nitrate (Al(NO_3_)_3_·6H_2_O), and potassium hydroxide (KOH). All chemical reagents were used as received without further purification. Bi(NO_3_)_3_·5H_2_O and Fe(NO_3_)_3_·9H_2_O were used as the source materials, while Al(NO_3_)_3_·6H_2_O and KOH were used as additives. Deionized water was used to make all aqueous solutions. A typical run for preparing BFO powders is as follows: The 20 mL each of Bi(NO_3_)_3_·5H_2_O, Fe(NO_3_)_3_·9H_2_O, and Al(NO_3_)_3_·6H_2_O were put into an 80-mL stainless steel autoclave and mixed well. After then, an appropriate amount of KOH solution was slowly dripped to the previous mixture solution until 65–80% of its volume was filled, which was next transferred into the strong magnetic stirring apparatus to stir for 2~3 h at 80 °C to get a clear solution. According to the method procedure, the obtained dark brown solution was transferred into Teflon-lined stainless steel autoclave. The hydrothermal treatment was carried out at a temperature of 200 °C for 10 h under autogenous pressure. The heating rate was 2 °C/min. After the hydrothermal reaction was complete, the resultant products were cooled down to RT naturally. Subsequently, the resultant powders were collected and washed several times with acetone, deionized water, and ethanol until the pH value of the solutions reached 7. Finally, the BFA_*x*_O powders were placed in a thermostat drying oven for 6 h at 70 °C, and then dried for further characterization. We have prepared four sets of samples of BFA_*x*_O by varying the concentration of Al(NO_3_)_3_·6H_2_O from 0–0.1 M.

The crystal structure of BFA_*x*_O samples was determined by X-ray diffraction (XRD, Mac Science M18XHF22-SRA). Raman spectroscopy (Renishaw InVia Reflex) with radiation from an Ar+ laser was employed to determine the structural properties of the powders at RT. XAFS data were collected in the transmission mode at various concentrations at the beamline 1W2B of the Beijing Synchrotron Radiation Facility (BSRF), China. Fe *K*-edge spectrum with an energy resolution of Δ*E*/*E*:2 × 10^−4^ and Bi *L*_3_-edge spectrum with an energy resolution of Δ*E*/*E*:1 × 10^−4^ were measured for the BFA_*x*_O samples at RT. In order to obtain best XAFS data, BFA_*x*_O powders were ground in an agate mortar, then mixed with BN and finally pressed into pellets. The background correction, normalization, and pre-edge and post-edge region of the absorption spectrum were fitted by ATHENA, a software for XAFS data processing, within the IFEFFIT program [33]. The *E*_o_ value was determined by the maximum in the first derivative in the edge region. We extracted the *χ*(*k*) profile in the *k* space of 0–12 Å^−1^. The *k*^3^ × *χ*(*k*) profile was Fourier transformed to the *R* space of 0–8 Å, by using the Hanning window function. The Fe_2_O_3_ and Bi_2_O_3_ were measured as the reference compounds. The optical properties of the powders were evaluated using ultraviolet-visible spectrophotometer (UV-Vis, UV 3900H). In this work, the investigation is limited to the low doping concentration of 0 ≤ *x* ≤ 0.1.

## Results and Discussion

**Fig. 1:**
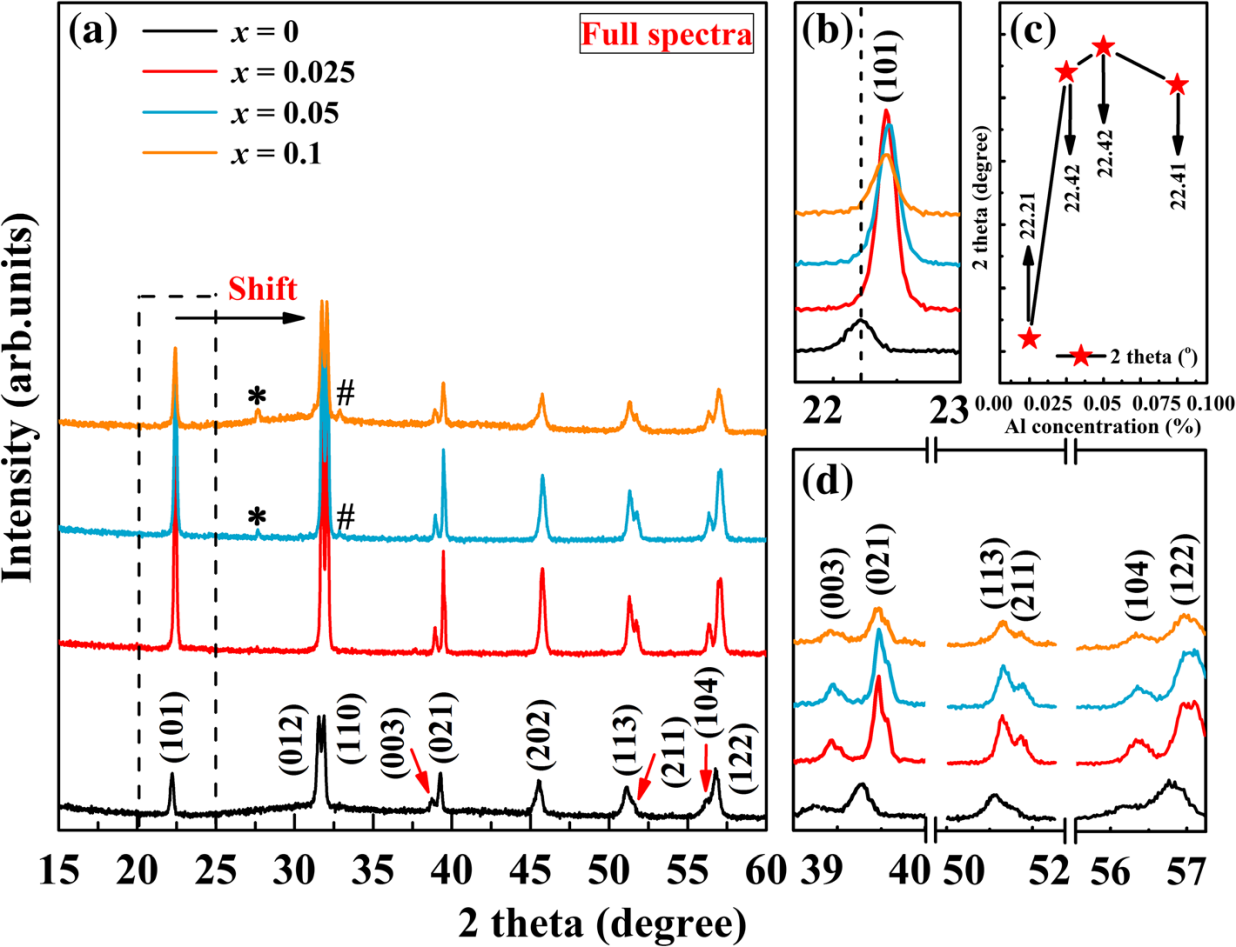
**a** XRD patterns of BFA_*x*_O (0 ≤ *x* ≤ 0.1). **b** Enlarged view of the XRD patterns in the range of 21–24°. **c** The (101) peak position as a function of Al concentration. **d** Magnified XRD patterns in the vicinity of 37–40°, 50–52°, and 55–57°

The XRD patterns of the undoped BFO (*x* = 0) and BFA_*x*_O powders, which scanned from 2*θ* value of 15–60°, are shown in Fig. 1. The full spectra of XRD patterns in Fig. 1a indicate that all samples can be identified as the standard diffraction data of the corresponding rhombohedral distorted perovskite structure (JCPDS Card File No. 20-0169, space group: *R3c*). It can also be seen that all samples exhibit neat diffraction patterns with a small amount of secondary phases. Traces of a secondary phase can be seen at 27.6° and 32.8° (marked “*” for Bi_2_Fe_4_O_9_ and “#”for Bi_25_FeO_40_) for *x* = 0.5 and *x* = 0.1 samples which may be resulting from the volatilization nature of Bi at the high sintering temperature [34]. This is often observed in the BFO powders synthesized by different routes [35–37]. Secondary phases are found to increase continuously at the high doping value. Therefore, B-site Al substitution for Fe cannot promote the pure phase of BFO; however, the electrical properties of samples would not be affected. From Fig. 1a, it can be seen that all diffraction peaks for the doped samples first shift to higher 2*θ* values with Al doping. For clarity, a part of XRD patterns in the 2*θ* region of 21 to 24° are amplified in Fig. 1b. From this enlarged view of the diffraction peaks, it can be observed that the (101) diffraction peak has an obvious shift toward high 2*θ* values with respect to undoped BFO, which confirms that Al is successfully doped into B-site of BFO. In analogy with undoped BFO, the (101) diffraction peak for the doped samples undergoes a shift in higher 2*θ* values first then small shift in lower 2*θ* values when *x* = 0.1 (surrounded by a dash line), as shown in Fig. 1c. Magnified XRD patterns in the vicinities of 37–40°, 50–52°, and 55–57° are given in Fig. 1d. There are some twin peaks, viz., (003) and (021), (113) and (211), (104) and (122), in the XRD spectrum. With the increase of Al content, the intensity of these peaks first increases then decreases when *x* reaches 0.1. As we know, the intensity of the peaks is usually related to the crystallinity. The decrease of the diffraction peaks indicates that the crystallinity of BFA_*x*_O decreases. Since Al^3+^ (0.51 Å) has a smaller ionic radius than Fe^3+^ (0.65 Å), it is easily incorporated into the BFO lattice when the doping amount is small, but excessive doping ion makes the BFO lattice unstable. The reduced crystallinity may be due to the fact that Al favors the creation of more nucleation sites which, in turn, inhibit the growth of crystal grains. Decreased crystallinity has also been found in other Al-doped BFO [28, 30]. On the other hand, this can occur if there is a creation of oxygen vacancies and transformation of some Fe^3+^ to Fe^2+^ due to the charge imbalance created in the system by Al^3+^ substitution. Similar phenomenon has been observed in Sr-doped BFO [38]. The shift in the diffraction peaks might be ascribed to the unit cell contraction, because of the small ionic radius of Al in comparison with Fe^3+^. The results of XRD show that the trivalent Al^3+^ substitution in BFO does not lead to observable structural transformation.

**Fig. 2:**
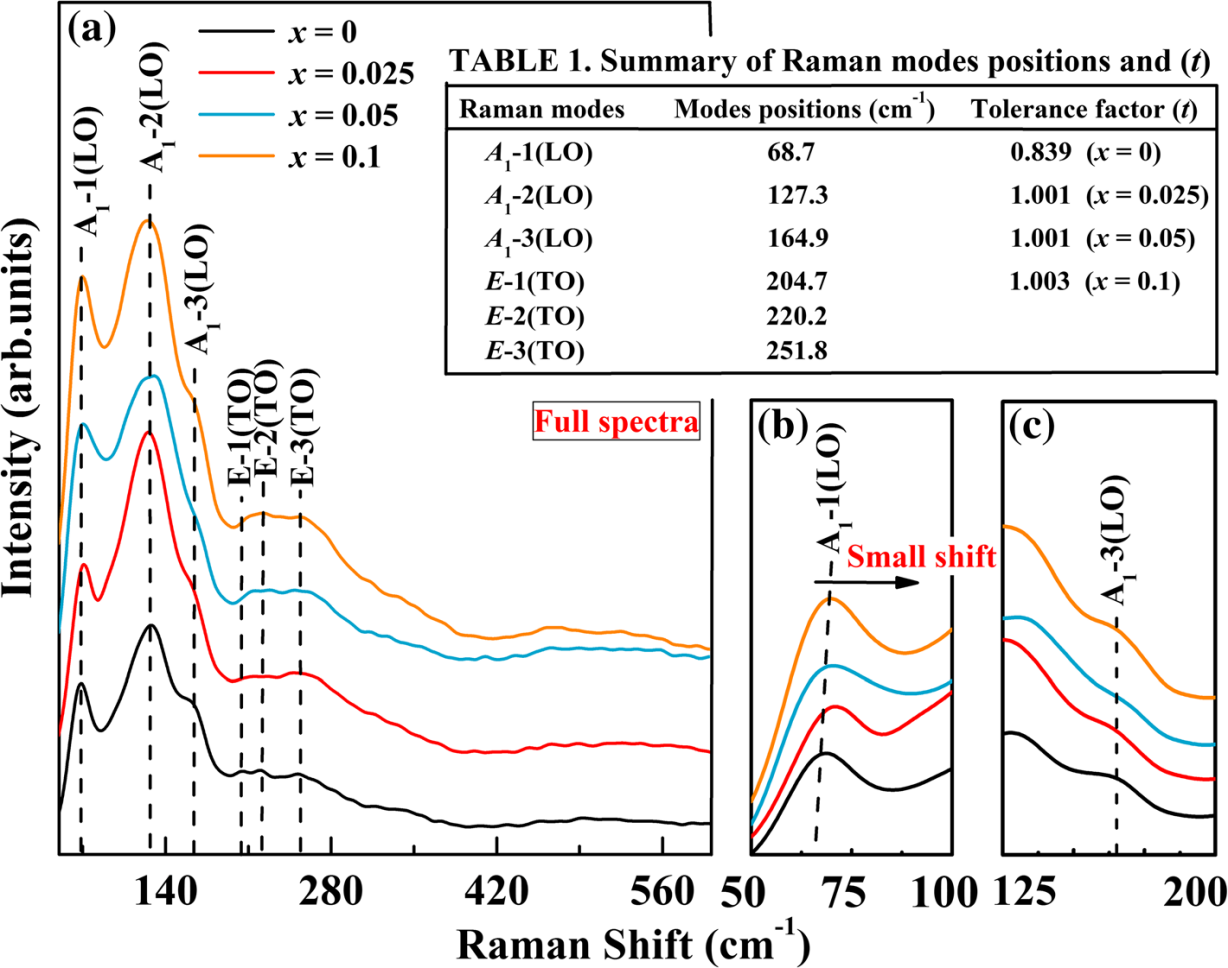
**a** Raman spectrum of BFA_*x*_O (0 ≤ *x* ≤ 0.1). **b** Enlarged Raman spectrum in the ranges of 50–100 cm^−1^ and **c** 125–200 cm^−1^

The structure confirmed by XRD can also be characterized by the position and intensity of Raman active modes. Raman spectrum is sensitive to the atomic displacement and distribution. The Raman scattering spectra of the undoped BFO and BFA_*x*_O powders are given in Fig. 2. On the basis of group theory, the 13 optical-phonon Raman active modes and 5 inactive modes (i.e., 5*A*_2_) of BFO are predicted for the rhombohedral distortion perovskites with space group *R3c* [39]. The theoretical analysis of the lattice vibrations in the perovskite *R3c* structure is as follows:

1 $$ {\varGamma}_{R3c}=4{A}_1\left(z,\kern0.5em {x}^2,{y}^2,\kern0.5em {z}^2\right)+5{A}_2\left(-\right)+9E\left(x,\kern0.5em y,\kern0.5em {x}^2-{y}^2,\kern0.5em xy,\kern0.5em xz,\kern0.5em y\right) $$

where *A* is longitudinal optical (LO) mode and *E* is the transverse optical mode (TO). In the full spectra shown in Fig. 2a, the number of clearly seen Raman active modes at RT is much less than predicted. In this work, we have observed six Raman active modes (3*A*_1_(LO) + 3*E*(TO)) for BFA_*x*_O powders. This may be due to the accidental degeneracies between bands in the spectra and the inability to distinguish weak bands from background noise [40], or dielectric leakage in the sample. For the undoped BFO, the strong and wide peaks at 68.7 cm^−1^, 127.3 cm^−1^, and 164.9 cm^−1^ were assigned to *A*_1_-1(LO), *A*_1_-2(LO), and *A*_1_-3(LO) modes, respectively. Peaks at 204.7 cm^−1^, 220.2 cm^−1^, and 251.8 cm^−1^ were assigned to *E*-1(TO), *E*-2(TO), and *E*-3(TO) modes, respectively (see Table 1 in Fig. 2). From the spectra, it can be clearly seen that these *E*(TO) modes are invisible. It is known that *A*_1_-1(LO) modes are attributed to the Bi-O bonds, while the tilt *A*_1_-3(LO) modes are viewed as the FeO_6_ octahedron. *E*(TO) modes are assigned to the Fe-O vibration [41]. The Raman result confirms that the prepared undoped BFO belongs to the rhombohedral distorted perovskite structure with space group *R3c*. It is noted that all four samples show similar Raman pattern and vibration modes. This indicated the same rhombohedral *R3c* space group, but their intensities and frequencies were somewhat different. At the whole view of Raman pattern of BFA_*x*_O samples, the peak position of the *A*_1_-1(LO) modes is slightly shifted to a higher frequency and the peaks of *A*_1_-3(LO) modes were broadened, indicating that the dopant Al is going to the B-site of BFO. The intensities of the *E*-1(TO), *E*-2(TO), and *E*-3(TO) modes are found to be slightly increased for the doped samples. For clarity, the *A*_1_-1(LO) and *A*_1_-3(LO) modes in the region of 50–100 cm^−1^ and 125–200 cm^−1^ are shown in Fig. 2b and c. From this enlarged spectrum, it is clear that *A*_1_-1(LO) modes exhibit a small shift to higher frequencies with increasing Al content. Compared to those of the undoped BFO, the peaks for *A*_1_-3(LO) modes were broadened for the doped samples. The small shift of the *A*_1_-1(LO) modes, slight broadening of *A*_1_-3(LO) modes, and change of the intensities of some *E*(TO) modes may be related to the change of the Bi-O and Fe-O covalent bonds and a compressive stress in the Al-doped sample [42, 36]. On the other hand, it is possible that all the above changes could be due to the fact that the B-site Fe^3+^ ions have been partially substituted by Al^3+^ ions. Apparently, these Raman results are consistent with the XRD observations. The Goldschmidt tolerance factor (*t*) is widely used to assess the geometric stability and distortion of crystal structures [43], where *t* is defined by the ratio of three kinds of ionic radii, as follows:

2 $$ t=\frac{\left({r}_{\mathrm{A}}+{r}_{\mathrm{O}}\right)}{\sqrt{2}\ \left({r}_{\mathrm{B}}+{r}_{\mathrm{O}}\right)} $$

where *r*_A_ is the radius of Bi^3+^, *r*_B_ is the average radii of Fe^3+^ and Al, and *r*_O_ is the radius of O^2-^. However, the radii of Al and Fe^3+^ are 0.51 Å and 0.65 Å, while the Bi^3+^ and O^2-^ have the radii of 1.03 Å (according to Ref. [44]) and 1.38 Å, respectively. The *t* values for our studied perovskite composites BFA_*x*_O are found to be 0.839, 1.001, 1.001, and 1.003 for *x* = 0, 0.025, 0.05, and 0.1, respectively (see Table 1 in Fig. 2). The ideal ABO_3_ compounds adopt a cubic close-packed structure when the value of *t* is 1, while when *t* < 1 or > 1, a geometric strain arises [45, 46]. As the Al concentration increases, the average B-site ionic radius decreases, which leads to a further increase in the *t* value from 0.839 to 1.003. This may be due to the small change in a low symmetric state of BFO. It is well known that the B-site cation in perovskite is surrounded by six oxygen anions, and when replaced by smaller ions, the coordination distance will decrease. Hence, to clearly analyze the effects of the Al substitution, it is necessary to study the local electronic structure of the BFA_*x*_O samples.

**Fig. 3:**
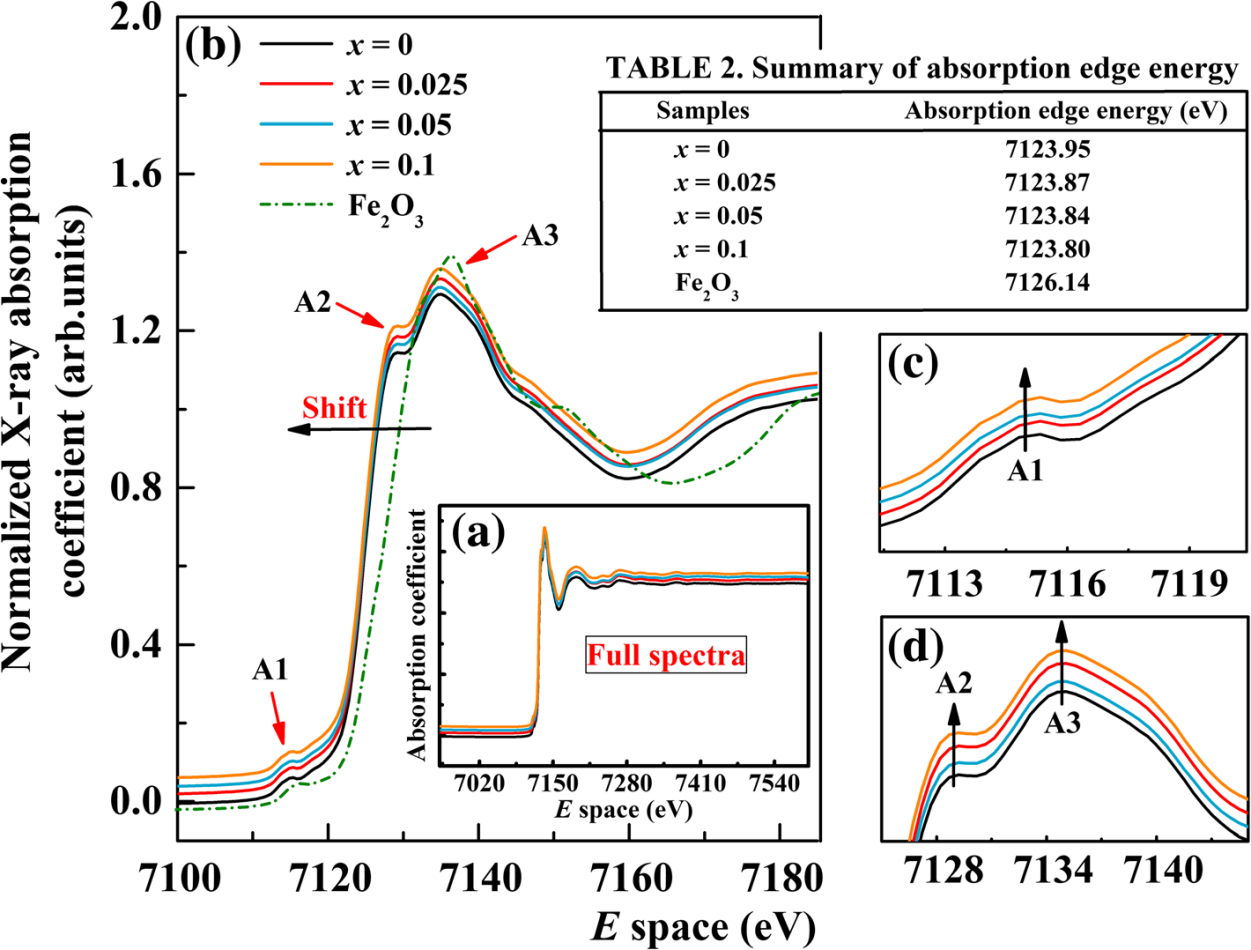
**a** Fe *K*-edge XANES spectra of BFA_*x*_O (0 ≤ *x* ≤ 0.1) and reference Fe_2_O_3_. **b** XANES spectrum in the range of 7100–7180 eV. **c** Enlarged view of the peak A. **d** Peak B and peak C

XAFS is divided into two types, i.e., X-ray absorption near edge structure (XANES) and extended X-ray absorption fine structure (EXAFS). In this measurement, when the X-ray penetrates through the slab with distance *x*, the intensity of the X-ray beam will be reduced to *I* = *I*_o_*e*^*-*μ*x*^. XAFS measures the absorption of X-rays as a function of X-ray energy *E*, that is, the X-ray absorption coefficient μ(*E*) = − *d* ln *I*/*dx* is determined from the decay in the X-ray beam intensity *I* with distance *x* [47]. The ratio of *I*/*I*_o_ is plotted as a function of *E* above the thresholds Fe *K*-edge (7112 eV) and Bi *L*_3_ (13,419 eV), which can provide important information about the shape of the μ(*E*). The electronic transitions shall be following the dipole selection rule. An X-ray absorbance μ(*ω*) can be obtained by Fermi’s golden rule, as follows:

3 $$ \upmu \left(\omega \right)\propto \sum \limits_f{\left|\left\langle f\left|D\right|\left.i\right\rangle \right.\right|}^2\ \delta \left\langle {E}_i-{E}_f+\omega \right\rangle $$

where |*i*⟩ is the initial state, |*f*⟩ is the final state, *D* is the dipole operator, *E*_*i*_ is the energy of |*i*⟩, *E*_*f*_ is the energy of |*f*⟩, and *ω* is the photon frequency. The XANES features contain useful information that are associated with the electronic structure of the absorber and local environment of an atomic structure. XANES function *χ*(*E*) is defined as follows:

4 $$ \chi (E)=\kern0.5em \left[\frac{\mu (E)-{\mu}_{\mathrm{o}}(E)}{\varDelta {\upmu}_{\mathrm{o}}}\right] $$

where μ_o_(*E*) is the smooth atomic-like background and Δμ_o_ is a normalization factor that arises from the net increase in the total atomic background absorption edge. The standard Eq. (4) was used for the background subtraction and removal during the processing of XANES data. Firstly, a smooth pre-edge function is subtracted to remove the background from the instruments. Secondly, μ(*E*) is normalized and then a smooth post-edge background function is subtracted from the μ(*E*) to obtain the μ_o_(*E*). Figure 3a shows the full spectra of the Fe *K*-edge XANES of an undoped BFO and BFA_*x*_O powders along with the reference compound Fe_2_O_3_ investigated in this work, while Fig. 3b reports the XANES spectrum in the energy range of 7100–7180 eV. From Fig. 3b, it can be seen that the shape of all spectra and peak positions is similar to each other. The absorption edges slightly shift toward lower energies with increasing Al concentration, which can be attributed to the chemical shift effect. The absorption edge energy shifts to lower energy with decreasing charge state, suggesting a complex bond configuration at the B-site. It is well known that edge shift could be used to obtain a mean oxidation state. The absorption edge energies for our studied samples BFA_*x*_O are found to be 7124.95 eV, 7123.87 eV, 7123.84 eV, and 7123.80 eV for *x* = 0, 0.025, 0.05, and 0.1, respectively. For the reference compound Fe_2_O_3_, the absorption edge energy is 7126.14 eV (see Table 2 in Fig. 3). The absorption edge of doped samples is lower than that of the Fe_2_O_3_ (Fe^3+^) reference compound. Increasing *x* values leads to the gradual shift of the absorption edge toward that of FeO (Fe^2+^) [48]. From this, we can note that BFA_*x*_O is a mixed-valent (Fe^3+^/Fe^2+^) system. In another aspect, three major features, pre-edge peak A1 and post-edge peaks A2 and A3, are observed in all four samples. The pre-edge peak A1, typical for BFO, corresponds to the electric quadrupole-forbidden transition from the O 1*s* level to Fe 3*d* ones, with a small admixture of Al 3*d* states. As the Al concentration increases, the intensity of the pre-edge peak A1 exhibits a small increase for the doped samples (see Fig. 3c). The post-edge peak A2 is attributed to the O 2*p* band transfer to the Fe 3*d* orbit, the so-called ligand-to-metal charge transfer process [49], whereas peak A3 is caused by the 1*s* to 4*p* dipole-allowed transition [50]. With increasing Al concentration, the intensity of these two post-edge peaks can be clearly seen to increase for the doped samples (see Fig. 3d). All the above changes in pre-edge and post-edge peak intensity can be understood in terms of competition between hybridization of Fe 3*d* and Al 3*d* with O 2*p* orbitals. Besides, after Al ion doping, there are more unoccupied 4*p* orbitals occurring in BFA_*x*_O. Except these, the whole spectra do not show significant changes and these results prove that Al ions are partially doped into B-site of BFA_*x*_O.

**Fig. 4:**
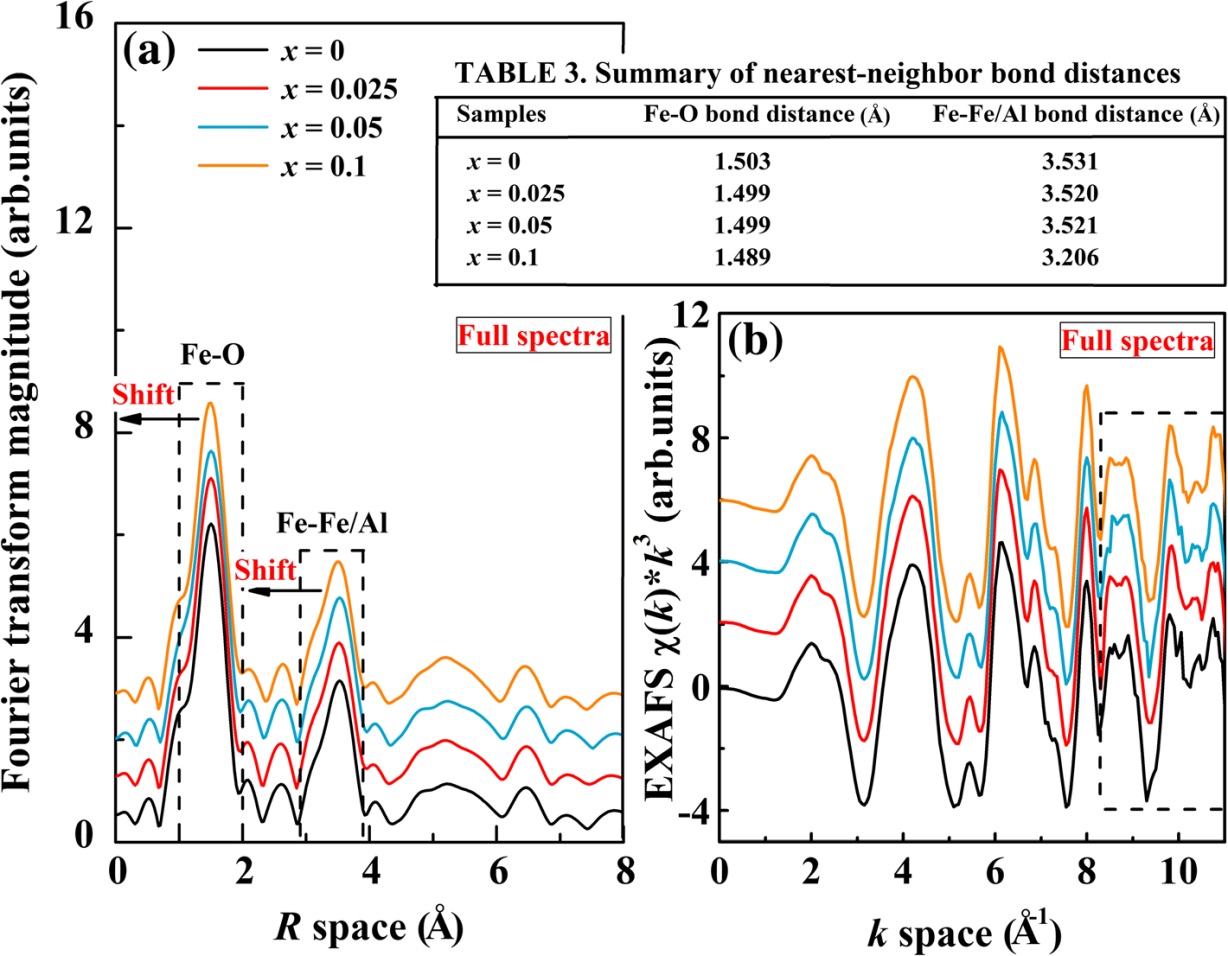
**a** Fourier transforms of Fe *K*-edge *k*^3^-weighted EXAFS data, for the BFA_*x*_O (0 ≤ *x* ≤ 0.1). **b** EXAFS *χ*(*k*) × *k*^3^ spectra of BFA_*x*_O (0 ≤ *x* ≤ 0.1). The spectra were aligned along the *Y*-axis for better comparison

The Fe-O, Fe-Fe/Al (i.e., Fe-O-Fe/Al), and Bi-O bond distributions are obtained by fitting the *k*^3^-weighted (*k*^3^ × *χ*(*k*)) raw data, as follows:

5 $$ k=\sqrt{\frac{2m\left(E-{E}_{\mathrm{o}}\right)}{\mathrm{\hbar}}} $$

where *E*_o_ is the absorption edge energy and *ħ* is the Planck constant. Further fitting (some approximations and oscillations) is made using the standard EXAFS equation, as follows:

6 $$ \chi (k)=\sum \limits_R{S}_{\mathrm{o}}^2{N}_{\mathrm{R}}\frac{\left|f(k)\right|}{k{R}^2}\sin \left(2 kR+2{\delta}_{\mathrm{c}}+\phi \right){e}^{\frac{-2R}{\lambda (k)}}{e}^{-2{\sigma}^2{k}^2} $$

where ${S}_{\mathrm{o}}^2$ ($0<{S}_{\mathrm{o}}^2<1$) is the reduction factor, *N*_R_ is the number of backscattering atoms at distance *R*, *f*(*k*) is the backscattering amplitude, *δ*_c_ is the phase shift from central, *ϕ* is the backscattering atoms, *λ* is the core-hole lifetime, *σ*^2^ is the Debye-Waller factor from multiple distances, and *k* is the photoelectron mean free path. The EXAFS region is typically referred to the energy range of 20–30 eV above the edge jump, which is sensitive to short-range order types, bond distances, and coordination numbers in materials. Although, Eq. (6) could give some information about the EXAFS approximations and oscillations, it is not a particularly convenient form for visualizing the information content of an EXAFS spectrum. Therefore, Fourier transformation can be used to decompose a *k* space signal into its different constituent frequencies [32]. Fourier transformation is a complex function of interatomic distance *R*, the amplitude of which is represented by the real function of *χ*(*R*). In this function, the position of peaks is related to bond distances and neighboring ions. Equation (6) can be transformed from *k* space to *R* space by Fourier transform, as follows:

7 $$ \chi (R)=\frac{1}{\sqrt{2\pi }}{\int}_{k_{\mathrm{min}}}^{k_{\mathrm{max}}}\omega (k){k}^n\chi {e}^{-2 ikR} dk $$

8 $$ \left\{\begin{array}{c}\genfrac{}{}{0pt}{}{\kern2.5em 0\kern7.5em k<{k}_{\mathrm{min}}\ }{\sin^2\left[\frac{\pi \left(k-{k}_{\mathrm{min}}\right)}{2\left({k}_2-{k}_{\mathrm{min}}\right)}\right]\kern3.25em {k}_{\mathrm{min}}<k<{k}_2}\\ {}\kern3.75em 1\kern5em {k}_2<k<{k}_3\\ {}\genfrac{}{}{0pt}{}{\cos^2\left[\frac{\pi \left(k-{k}_3\right)}{2\left({k}_{\mathrm{max}}-{k}_3\right)}\right]\ \kern2.75em {k}_3<k<{k}_{\mathrm{max}}}{\kern2em 0\kern7.5em k>{k}_{\mathrm{max}}}\end{array}\right. $$

where *k*_max_ and *k*_min_ are the maximum and minimum values of transformed *k* space, respectively, *χ*(R) is the Hanning window function, *ω*(*k*) is the Gaussian window function, and *k*^*n*^ is the weight factor (*n* = 0, 1, 2, 3). The *k*_2_ and *k*_3_ values for the BFA_*x*_O are 2 and 10, respectively, and the value of *n* is 2. Fourier transform of Fe *K*-edge EXAFS of the BFA_*x*_O samples is performed, as shown in Fig. 4a. The asymmetric peak (surrounded by the first dash line) centered at ~ 1.503 Å is identified as a Fe-O bond, owing to scattering from the oxygen anions. The second strong peak (surrounded by the second dash line) around ~ 3.527 Å is corresponding to the Fe-Fe/Al bonds, which can be explained by the scattering of oxygen anions from the next-to-nearest-neighboring Fe/Al atomic shells. The distance for the first and second coordination shells has been summarized in Table 3 in Fig. 4. From the peak position, compared with undoped BFO sample, Fe-O and Fe-Fe/Al bond tends to slightly shift toward smaller *R* values with increasing *x*. This indicates that doping by Al ions not only affects the nearest-neighbor local structure of central Fe atom but also affects the next-nearest coordination shells of the Fe atom. On the other hand, the shift of the Fe-O bond to smaller *R* values may result from that the radius of Al ions is smaller than that of Fe ions. This is consistent with the XRD data. The shift of Fe-Fe/Al to smaller *R* values also indicates that the average Fe-Fe/Al bond length (where the two Fe^3+^ ions are in the centers of neighboring oxygen octahedral) gradually becomes shorter and the bond angle is modified in the Al-doped samples. However, there is a small increase of peak intensity of the Fe-O distribution for the doped samples while the peak intensity of the Fe-Fe/Al distribution is almost not changed. This reveals that the iron-neighboring structure of Fe-O has been modified by Al doping. The shorter Fe-Fe/Al bonds in Al-doped samples can explain why the major peaks (101) in XRD shift to higher 2*θ* angles (see Fig. 1b). This indicates that the substitution of Al for Fe could affect oxygen octahedral, which further reduces the coordination distance between the two neighboring Fe atoms. Figure 4b shows the Fe *K*-edge EXAFS of the BFA_*x*_O samples processed on *k*^3^ × *χ*(*k*) oscillation with a *k* space of 0–10 Å^−1^. As can be seen, all the *k*^3^ × *χ*(*k*) spectra show similar patterns at the smaller *k* values but different at larger *k* values with some various noise (surrounded by the dash line). Al-doped samples show a broader *k*^2^× *χ*(*k*) spectrum than those of undoped BFO in the *k* space of 8.2–9.3 Å^−1^, implying an enhanced short-range structural disorder in BFA_*x*_O samples. The noises are observed in a *k* space of 10–10.4 Å^−1^. These changes indicated that the local structure of center atoms has changed due to B-site Al doping, similar to what was reported by Li et al. [51].

**Fig. 5:**
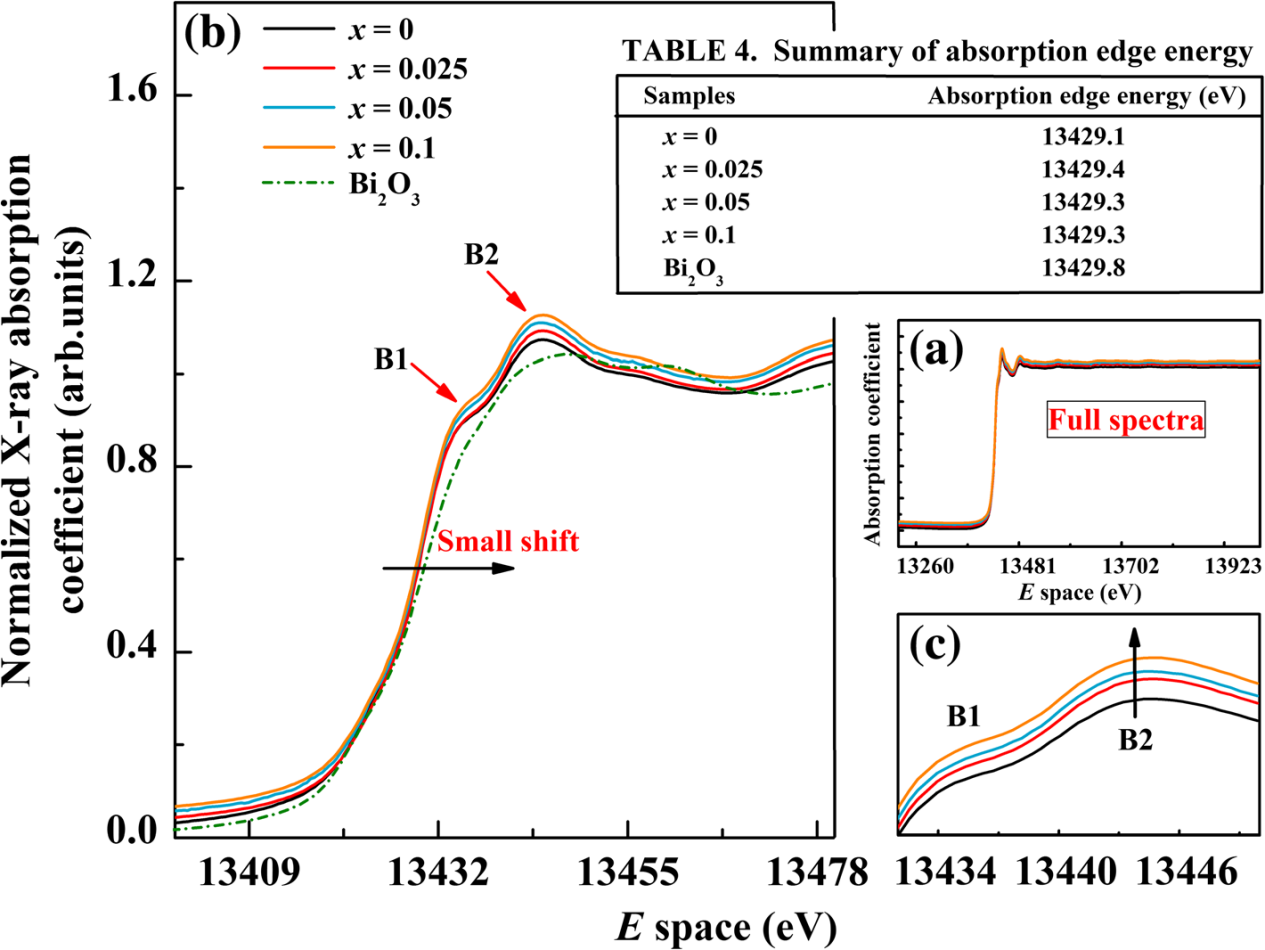
**a** Bi *L*_3_-edge XANES spectra of BFA_*x*_O (0 ≤ *x* ≤ 0.1) and reference Bi_2_O_3_. **b** XANES spectrum in the range of 13,400–13,480 eV. **c** Enlarged view of the peak D and peak E

The peak positions, intensities, and shapes of the line in the Bi *L*_3_-edge XANES spectrum are well known to depend on the local electronic structure of the Bi atoms, which could provide information on the Bi valence. The full spectra of Bi *L*_3_-edge XANES of undoped BFO and BFA_*x*_O samples are also shown in Fig. 5a, while Fig. 5b shows the Bi *L*_3_-edge XANES spectrum in the energy region of 13,400–13,480 eV. The analysis of these spectra helps to investigate the local electronic structure of Bi ions in the doped system. From Fig. 5b, it can be seen that the shape of all spectra are the same to each other and there is almost no change of absorption edge in the whole series. The absorption edge energies for our study are found to be 13,429.1 eV, 13,429.4 eV, 13,429.3 eV, 13,429.3 eV, and 13,429.8 eV for *x* = 0, 0.025, 0.05, and 0.1 and the reference compound Bi_2_O_3_, respectively (see Table 4 in Fig. 5). The absorption edges slightly shift toward higher energies with increasing Al concentration. The absorption edge in the Bi *L*_3_-edge of the BFA_*x*_O samples matches well with that of the reference compound Bi_2_O_3_, which indicates that the valence state of Bi ions in all the samples is in + 3 valence state. However, there are two post-edge peaks found in all samples and marked as B1 and B2, respectively. These two post-edge peaks are caused by the electric-forbidden transition from 2*p*_3/2_ level to the 6*d* ones. Compared with undoped BFO, the intensity of peak B2 can be clearly seen to increase for the doped samples (see Fig. 5c), which means the transition from 2*p*_3/2_ to 6*d* state increases, so does the energy of 6*d* state. Except these, there is no other significant change in the whole spectrum.

**Fig. 6:**
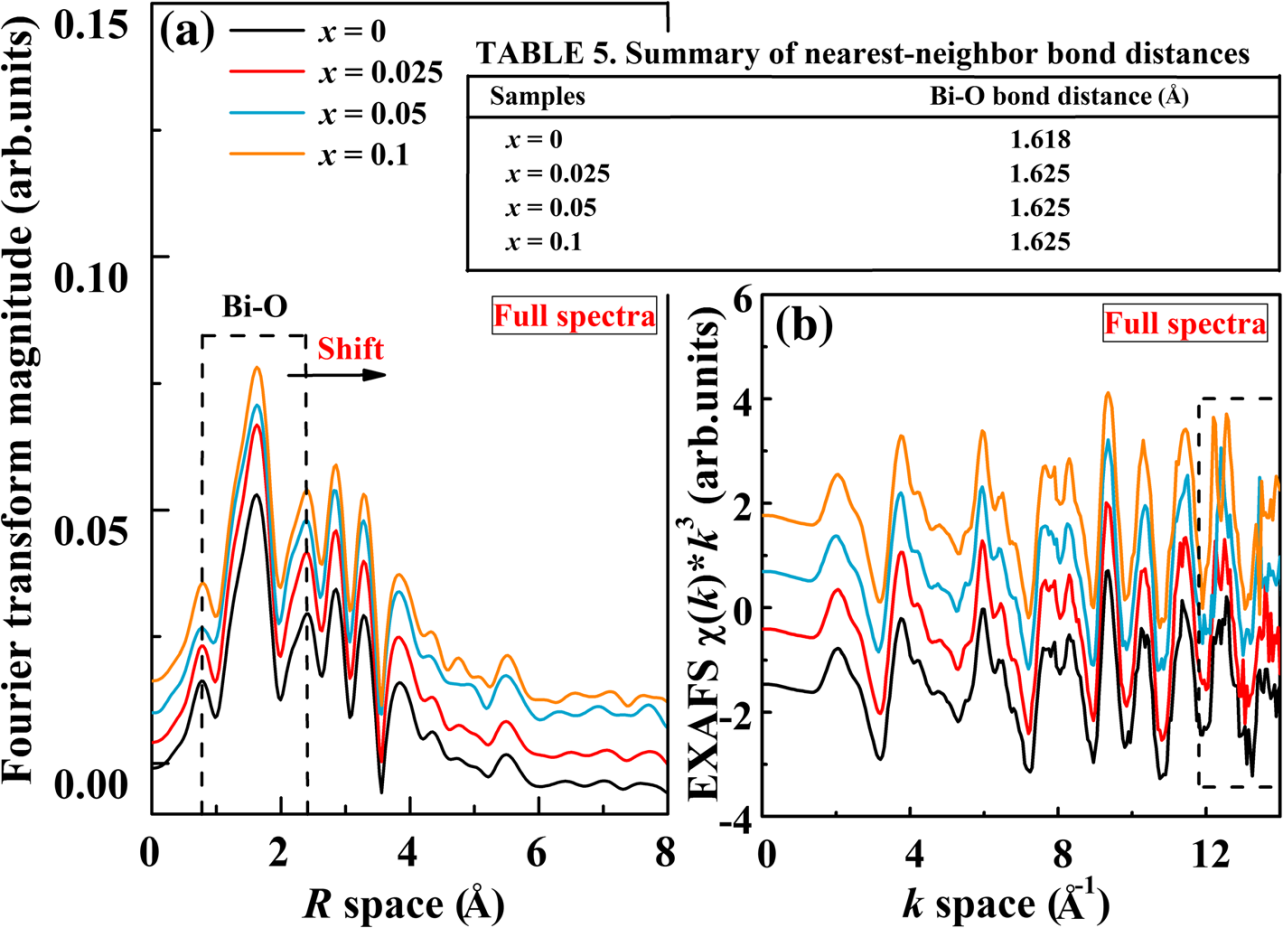
**a** Fourier transforms of Bi *L*_*3*_-edge *k*^3^-weighted EXAFS data, for the BFA_*x*_O (0 ≤ *x* ≤ 0.1). **b** EXAFS *χ*(*k*) × *k*^3^ spectra of BFA_*x*_O (0 ≤ *x* ≤ 0.1). The spectra were aligned along the *Y*-axis

Fourier transform of Bi *L*_3_-edge EXAFS radial distribution functions is performed, as shown in Fig. 6a. A high-intensity peak located at around 1.618 Å corresponds to the nearest Bi-O coordination shell (surrounded by the dash line), which is a result from scattering from the nearest-neighbor atomic shell of Bi, i.e., oxygen anions. However, the position of Bi-O bond shifts toward larger *R* values for the doped samples (see Table 5 in Fig. 6). This indicates that the substitution of Al for Fe could affect the nearest-neighbor local structure of the central Bi atom. It also indicates the extension of Bi-O bond length. The peak intensity of the Bi-O distribution exhibited a small increase with increasing Al content, which suggests that the iron-neighboring structure of Bi-O has changed. Figure 6b shows the Bi *L*_3_-edge *k*^3^ × *χ*(*k*) EXAFS spectra with a *k* space of 0–14 Å^−1^. From Fig. 6b, it can be seen that all the spectra shape shows similar patterns except some error noises. The error noises are observed in the *k* space of 12–14 Å^−1^ (surrounded by the dash line). This result may imply that the *k*^3^ × *χ*(*k*) EXAFS function of the center Bi atoms has changed with Al doping. This also suggests that the B-site Al substitution influences short-range structural disordering.

**Fig. 7:**
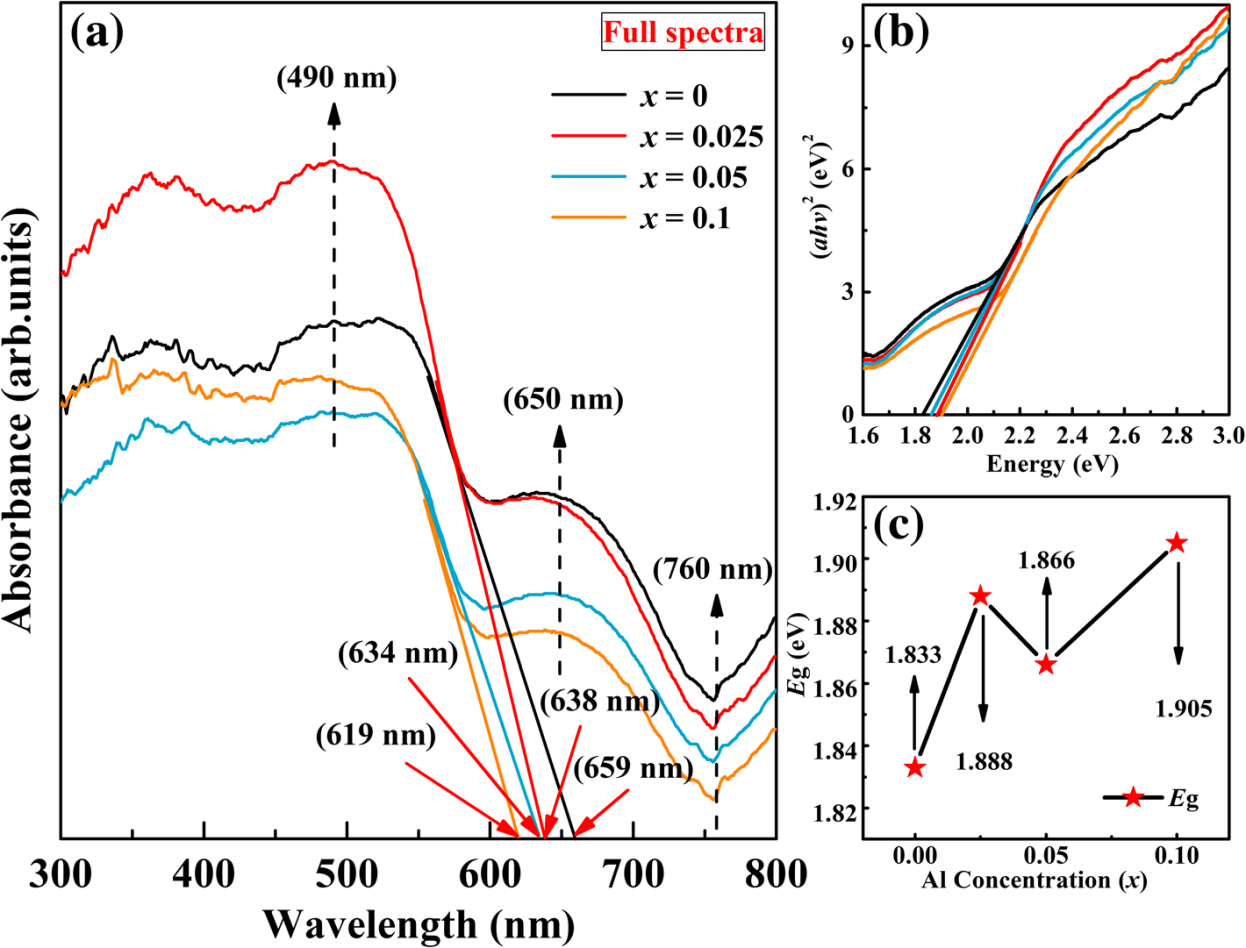
**a** UV-Vis absorption spectrum of BFA_*x*_O (0 ≤ *x* ≤ 0.1). **b** Plots of (*ahν*)^2^ vs. photon energy. **c** *E*g values as a function of Al concentration

The optical properties of the samples are studied by using RT UV-Vis, which is used to characterize the optical properties of the materials. The UV-Vis absorption spectra of undoped BFO and BFA_*x*_O samples in the wavelength of 300–800 nm are shown in Fig. 7a. As a result, the UV-Vis spectra of the undoped BFO and BFA_*x*_O samples show two absorption edges (marked by dashed arrows). One is a band around 650 nm, which is due to the metal-to-metal transition. The other is a band around 760 nm, which is caused by crystal field transition [52]. In addition, the strong absorption band is observed at about 490 nm (marked by dashed arrow), which is attributed to the electronic transition from O *2p* to Fe *3d* state in the BFO. These strong bands indicate that the BFO prepared by hydrothermal method could be a promising visible-light photocatalytic material. BFO is of direct transition with a value of *n* as 2. The absorption edge of the doped samples shifted from 659 to 619 nm, suggesting that the BFA_*x*_O powders absorb visible light in the wavelength range of 600–659 nm (see Fig. 7a). A similar blue-shift phenomenon was observed earlier in other element-doped BFO [53–55]. This blue shift in the absorption spectra of Al-doped samples in comparison with the undoped BFO shows that doping Al causes a change in the local structure for BFO. From Fig. 7a, one can see that the absorption spectra of the Al-doped samples exhibit a sharp increase around 490 nm and it suggests that all samples can absorb remarkable amounts of visible light. For the sample with *x* = 0.025, the absorption spectrum shows a sudden increase. It means that it has a wider absorption range than the other samples in this range of visible light. The optical band gap of the samples has been calculated by Tauc’s formula, as follows:

9 $$ ahm=A{\left( hm-E\mathrm{g}\right)}^n $$

where *a* is the absorption coefficient, *A* is the parameter*, h* is the Planck’s constant, *m* is the frequency of the incident photon, *E*g is the optical band gap, and *n* (for direct *n* = 2, for indirect *n* = 0.5) is a constant associated with different types of electronic transitions, as shown in Fig. 7b. The calculated *E*g values are found to be 1.833 eV, 1.888 eV, 1.866 eV, and 1.905 eV for *x* = 0, *x* = 0.025, *x* = 0.05, and *x* = 0.1 samples, respectively. It is easy to see that the band gap increases with the substitution ratio, as shown in Fig. 7c. The increase in the band gap is attributed to the doping effect. The *E*g value for the undoped BFO is about 1.833 eV, which is lower than the previous reports [56, 57].

## Conclusion

In summary, the BFA_*x*_O (*x* = 0, 0.025, 0.05, and 0.1) multiferroic powder samples were successfully synthesized via hydrothermal route. Effects of Al substitution on the structural, electrical, and optical properties of the samples were studied. The structural study reveals that Al-doped BiFeO_3_ shows the existence of secondary phases and lattice contraction due to lower ionic radii of Al doped into B-site, which still retains its rhombohedral *R3c* perovskite structure. Raman scattering measurement infers six Raman active phonon modes, which further confirms the result of XRD. XAFS studies on the Fe *K*-edge and B *L*_3_-edge of the BFA_*x*_O samples and of the reference compounds Fe_2_O_3_ and Bi_2_O_3_ were performed, and the obtained results were compared in order to determine the valance states of Fe and Bi ions in the system. The Fe *K*-edge XAFS results revealed that BFA_*x*_O is a mixed-valent (Fe^3+^/Fe^2+^) system. The results of Fe *K*-edge XAFS also illustrate a competition between the Fe 3*d* and Al 3*d* orbitals on hybridization with the O 2*p* and occurrence of the more 4*p* orbitals with Al doping. Besides, Al ion doping affects both the nearest-neighbor and next-nearest coordination shells of the Fe atom. The B *L*_3_-edge XAFS results indicate that valence states of Bi ions in all the samples are in + 3 and the transition from 2*p*_3/2_ to 6*d* state and the energy of 6*d* state increases. Substitution Al for Fe could affect the nearest-neighbor local structure of central Bi atom. The BFA_*x*_O prepared by hydrothermal method could be an appropriate visible-light photocatalytic material due to a strong absorption band in the visible region.

## Abbreviations

AFM: Antiferromagnetic
BFA_*x*_O: BiFe_1-*x*_Al_*x*_O_3_
BFO: BiFeO_3_
EXAFS: X-ray absorption fine structure
FE: Ferroelectric
FM: Ferromagnetic
RT: Room temperature
UV-Vis: Ultraviolet-visible
XAFS: X-ray absorption fine structure
XANES: X-ray absorption near edge structure
XRD: X-ray diffraction
